# Impacts of climate variability and land use on the blue and green water resources in a subtropical basin of China

**DOI:** 10.1038/s41598-022-21880-3

**Published:** 2022-12-05

**Authors:** Meibing Liu, Di Wang, Xingwei Chen, Ying Chen, Lu Gao, Haijun Deng

**Affiliations:** 1 State Key Laboratory for Subtropical Mountain Ecology of the Ministry of Science and Technology and Fujian Province, Fujian Normal University, Fuzhou, 350007 China; 2 School of Geographical Sciences, Fujian Normal University, No.8 Shangsan Rd, Cangshan District, Fuzhou, 350007 China; 3 Fujian Provincial Engineering Research Center for Monitoring and Assessing Terrestrial Disasters, Fujian Normal University, Fuzhou, 350007 China

**Keywords:** Climate sciences, Hydrology

## Abstract

Water scarcity has become a global severe challenge over the past few decades. Quantifying the impact of climate variability and land use on water resource availability is crucial for integrated water resource management. Many studies have focused on blue water but ignored green water which is important in the terrestrial ecosystem, especially on different temporal scales. In this study, we selected the Shanmei Reservoir, the most import drinking water resource for a rapidly development city of Southeast China, as a case for analysis of these impacts for the entire basin. We adopted the Soil and Water Assessment Tool (SWAT) to investigate the spatial and temporal distributions of blue water (BW), green water flow (GWF) and green water storage (GWS) in the Shanmei Reservoir Basin (SRB). The results of the blue and green water components (BW and GW) revealed that SRB is dominated by BW, accounting for 52.6% of the total water resources, while GW accounted for 47.4%. There was an insignificant upward trend of BW and a significant upward trend of GWF, with a tendency rate of 1.125 mm a^−1^. Precipitation was the key factor affecting BW on annual and monthly scales. The GWF was more sensitive to temperature at both the annual and monthly scales. The GWS was significantly correlated with precipitation at the monthly scale, while insignificant correlation occurred at the annual scale. The spatial distribution of BW was largely dominated by precipitation, and land-use types led to the differentiation of GW. It indicates that the BW of paddy fields is greater than that of forests, while the GWS of forests is greater than that of orchards and rainfed croplands.

## Introduction

Freshwater resources are very important for maintaining the ecological balance and sustainable development of human society. Over the past few decades, water scarcity has become a severe global challenge^1–3^. Climate variability affects precipitation and temperature and alters the spatial–temporal distribution of water resources^4^, exacerbating the contradiction between the supply and demand of water resources. Human activities, such as water consumption and land use change, have altered evapotranspiration, runoff, infiltration, and soil water redistribution and changed the water volume distribution in the basin^5,6^. Therefore, it is necessary to understand the impacts of climate variability and land-use patterns on regional water resources and improve the effective utilization rate of available water resources to realize the sustainable development of water resources and the ecological environment^7^.

Falkenmark^8^ introduced the concepts of blue water (BW) and green water (GW). BW is defined as surface water and groundwater that can be directly used for industrial production and agricultural irrigation. This has always been the focus of traditional water resource management and water assessment. GW refers to the water stored in the soil and subsequently fed back to the atmosphere through crop evapotranspiration, including the green water flow (GWF) and green water storage (GWS). BW is critical for social and economic development, whereas GW is extremely important for ecosystem health and food production^9,10^. On the global scale, approximately two-thirds of water resources are stored as GW^11,12^. GW is the main water resource for rain-fed agriculture of global farmlands and contributes significantly more than BW. More than 87% of the global water consumption of humans, especially agricultural products, comprises GW^13,14^. However, most studies have focused on evaluating BW while ignoring the spatial–temporal variations of GW.

Climate variability significantly affects the temporal distribution of BW and GW resources^15–17^. For example, the decline in BW resources occurs mainly due to a decrease in precipitation, while an increase in temperature promotes evapotranspiration and leads to an increase in GWF^18,19^. The spatial distribution of BW resources is mainly controlled by precipitation^20,21^, while that of GW is not only affected by climate variability but also related to the underlying surface properties. The spatial differentiation of BW and GW is significant under different land cover types, which depend on the evapotranspiration capacity of the underlying surface^22,23^. Zhao et al.^24^ indicated that the amount of BW and GW in farmland was higher than that of forest and grass; the decrease in farmland and the increase in forest and grassland reduced BW and GWF, while augmenting the GWS. Liang et al.^15^ found that urban expansion increases impervious surfaces, thus increasing surface runoff and reducing surface permeability and soil water content^22^. Li et al.^25^ concluded that land use has little impact on the total evapotranspiration of the basin, while having a significant impact on its spatial distribution; they also discovered that GW resources in some subbasins will vary significantly with considerable changes in land use and cover. However, other studies have also found that land-use change has little impact on GW, and its impact on BW is greater than that on GW^25,26^. The effects of climate variability and land use on BW and GW resources have not been fully determined. Furthermore, most studies on BW and GW resources used annual average values; however, they did not conduct evaluations on finer temporal scales (such as seasonal and monthly scales), which may improve our understanding of the drivers of BW and GW resources.

The Shanmei Reservoir, located in the south-eastern coastal area of China, is the most important drinking water resource in Quanzhou City, with a developed economy and a large population. The temporal and spatial distribution of water resources in the Shanmei Reservoir Basin (SRB) is rather uneven, which results in serious regional and seasonal water shortage problems. Therefore, the objectives of our study were to: (1) reveal the evolutionary characteristics of climate (precipitation and temperature) variability in the basin; (2) analyze the temporal and spatial distribution of BW and GW resources in the SRB using the Soil and Water Assessment Tool (SWAT) model; and (3) estimate the impacts of climate variability and land use on BW and GW resources within the basin. The results of the paper will be useful to understand the spatio-temporal pattern of water distribution in a reservoir basin which has an important significance in rationally utilizing water resources and maintaining the health of water ecosystem in the SRB.

## Materials and methods

### Study area

**Figure 1:**
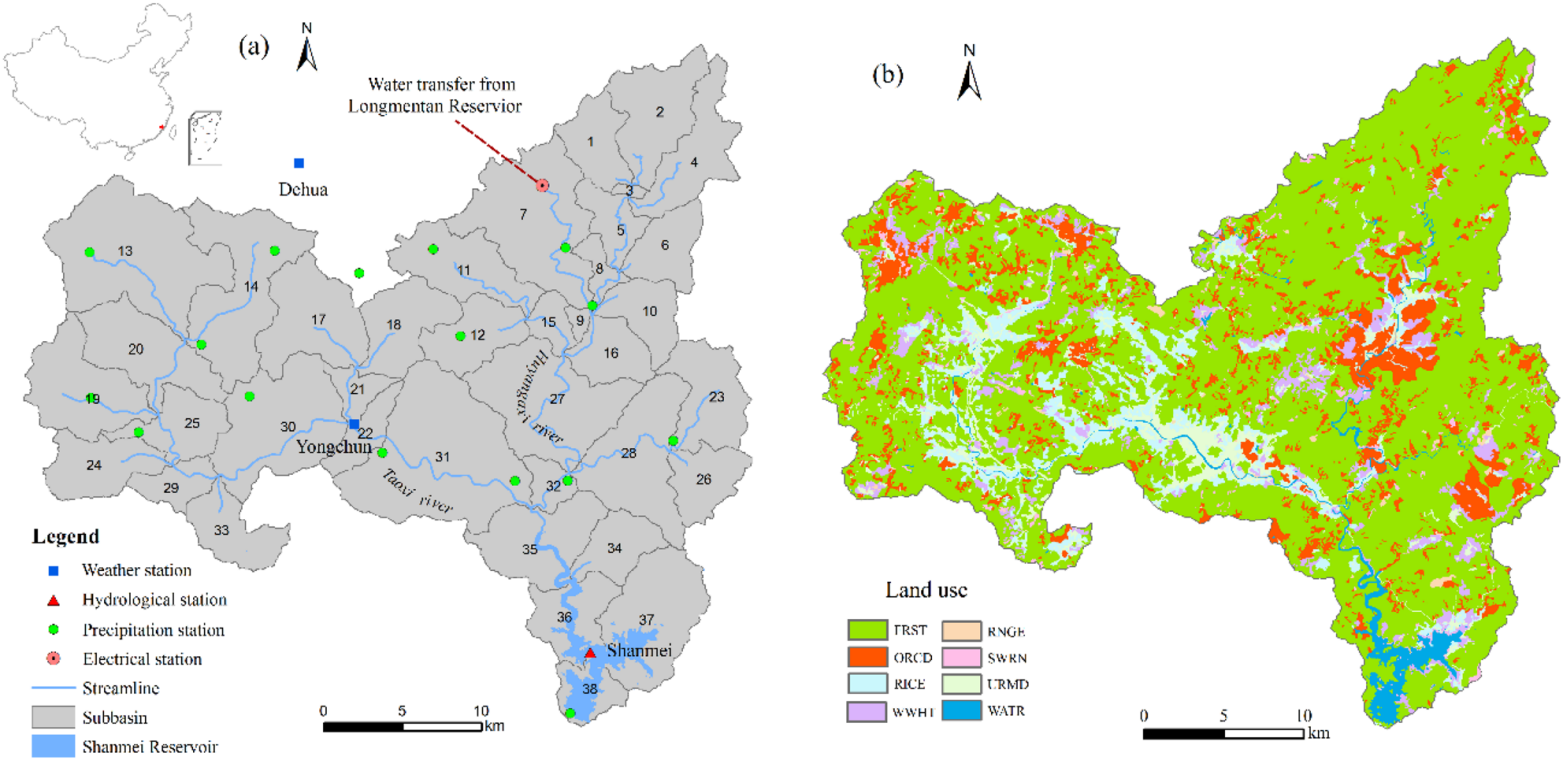
(**a**) Distributions of subbasins and river lines in the Shanmei Reservoir Basin (SRB); (**b**) distributions of the land use types in the SRB (Land use classes correspond SWAT codes to RICE (paddy field), WWHT (rainfed cropland), FRST (forest), ORCD (orchard), RNGE (grassland), URMD (medium density residential), WATR (water), and SWRN (bare land)).

The Shanmei Reservoir is a large reservoir with the functions of water supply, irrigation, flood control, and power generation. It undertakes the domestic and production water demand of four million residents downstream of the reservoir. The Shanmei Reservoir has a total storage capacity of 6.55 × 10^8^ m^3^ and effective storage of 4.72 × 10^8^ m^3^, with the normal water level reaching 96.48 m. It has a drainage area of 1023 km^2^, with the average annual runoff into the reservoir reaching 14.00 × 10^8^ m^3^. The SRB is drained by inter-basin Longmentan water transfer and two rivers (Taoxi River and Huyangxi River) (Fig. 1a). The basin has a subtropical monsoonal climate. The annual average temperature is approximately 20.90 °C, and the average annual precipitation is 1800 mm. The precipitation from April to October accounts for 84% of the annual precipitation, while that from November to March accounted for 16%. Precipitation exhibits significant interannual variability, with the precipitation amount in wet years being more than 2 times of that in dry years. Furthermore, the spatial distribution of water resources is very uneven; that is, 70% of water resources are concentrated in relatively backward mountainous areas. In densely populated coastal economically developed areas, capital water resources reach only 218 m^3^. Available water resources are rather limited, leading to serious water shortage problems and affecting the sustainable development of the social economy.

### SWAT model

The SWAT model is a physically based semi-distributed hydrological model. It exhibits great advantages in evaluating the impacts of climate variability and human activities on water quantity and quality^21,27,28^. Our study used the SWAT model to calculate BW and GW. The hydrologic cycle equation in the land phase as simulated by SWAT is based on the following governing water balance Eq. ^27^.

1 $$SW_{t} = SW_{O} + \sum\nolimits_{i = 1}^{t} {\left( {R_{day} - Q_{sur} - ET - W_{seep} - Q_{gw} } \right)}$$

where, SW_t_ is the final soil water content (mm); SW_o_ is the previous soil water (mm); t is the step of time (d); R_day_ is the amount of precipitation on day i (mm), Q_surf_ is the amount of surface runoff on day i (mm), ET is the amount of evapotranspiration on day i (mm), W_seep_ is the amount of water entering the vadose zone from the soil profile on day i (mm), and Q_gw_ is the amount of the return flows on day i (mm).

The input data for the SWAT model included topography, land use type, soil data, and weather data. The topography was represented by 30 × 30 m elevation raster, which was obtained from the International Scientific Data Platform of the Chinese Academy of Sciences (http://datamiffor.csdb.cn/admin/datademMain/jsp). The soil information map (1:500,000) from the Soil Fertilizer Laboratory of Fujian Province was used to identify ten soil types in the watershed. The land use map was interpreted using Landsat Thematic Mapper (TM) remote sensing images from 2006. Land use was classified into eight types: paddy fields, rainfed croplands, forests, orchards, grasslands, medium-density residential areas, water bodies, and bare land (Fig. 1b). The SRB was first divided into 38 sub-basins and then defined as 297 hydrology response units (HRUs) based on the land use type and soil information, with a threshold area of 1500 ha (Fig. 1a). Climatic data were obtained from Yongchun and Dehua meteorological stations, which provided daily average precipitation, relative humidity, wind speed, and daily maximum and minimum temperatures. The solar radiation data were calculated using the weather generator in the SWAT. Sixteen precipitation stations within the basin provided daily precipitation data for 31 years (1980–2010).

Evapotranspiration (ET) has a major role in estimation of green water. SWAT model calculates ET in the forms of potential ET (PET) and actual ET (AET). The model offers different options to estimate PET including the Hargreaves, Priestley Taylor and Penman-Monteith^27^. Here we chose to apply the Penman–Monteith (PM) method for computing PET in our study area.

We evaluated the performance of the daily runoff simulated by SWAT using a five-year period (2001–2005) for calibration and another five-year period (2006–2010) for validation. The simulated daily runoff performed well with a Nash–Sutcliffe efficiency (E_NS_) of 0.88 for the calibration and 0.87 for the validation. The *E*_NS_ values of monthly runoff were 0.93 and 0.96 for the calibration and validation, respectively. The statistical results of the *E*_NS_ indicate that the model performance was satisfactory^29^. Therefore, the SWAT model can simulate runoff within the SRB. The specific calibration details of the model are provided in our previous work^28^.

### BW and GW calculations

BW of each subbasin was simulated by combining both the water yield (WYLD, mm) and the storage of groundwater (∆GW, mm). WYLD represents the water flowing from the HRU to the main channel (SWAT output.sub), and △GW represents the water entering the aquifer (GW_RCHG, mm) minus the water entering the main channel from the aquifer (GW_Q, mm). The data of GW_RCHG and GW_Q are both from output.hru file of SWAT^30^.

GW can be divided into GWF and GWS. GWF is represented by the actual evapotranspiration (ET, mm), and GWS is the soil moisture content (SW, mm)^31^. The data of ET and SW are both from output.sub file of SWAT.

### Mann–Kendall method

The Mann–Kendall (M–K) nonparametric trend test has been widely used in long-term trend analysis on meteorological and hydrological time series^18,32^. The M–K test detected the trend changes in the annual average temperature, precipitation, BW, and GW in the SRB.

## Results

### Trend analyses of climate variability

**Figure 2:**
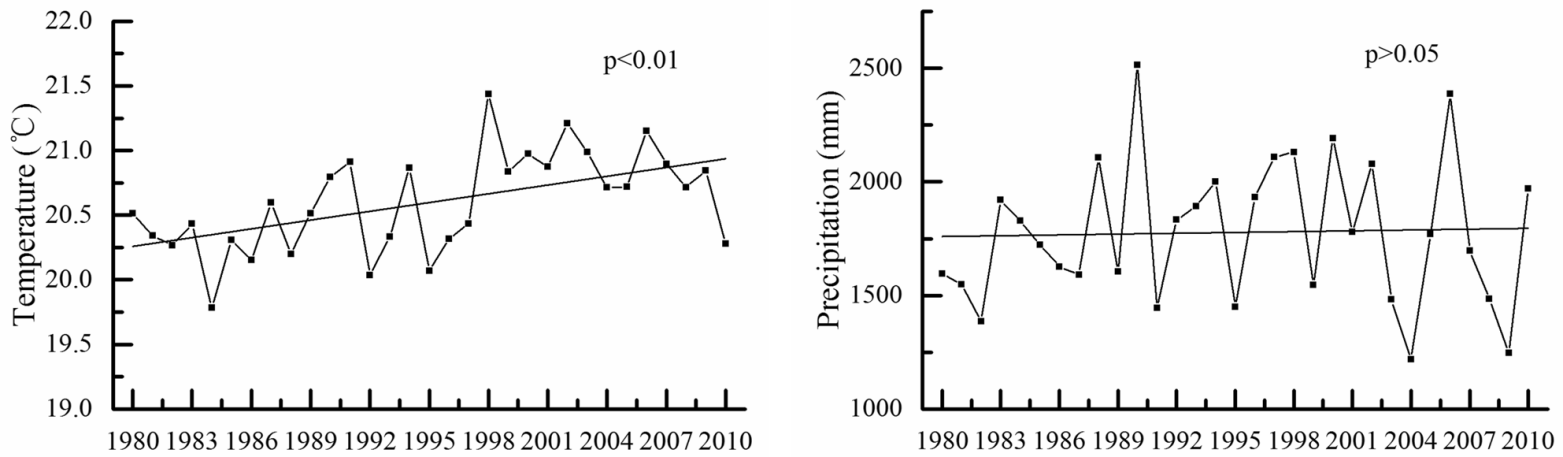
The trend analyses of temperature and precipitation in SRW for 1980–2010.

The annual average temperature in the SRB showed an increasing trend from 1980 to 2010 (*p* < 0.01) (Fig. 2). There was a short decreasing trend in the early 1990s, while a sudden change occurred around 1994; however, the annual average temperature began to rise significantly after 1996. Meanwhile, the mean precipitation fluctuated dramatically during 1980–2010. The trend rate and trend statistic (Z) values for annual temperature and precipitation from 1980 to 2010 are displayed in Table 1. In summary, the average temperature in the basin increased steadily, with a significant increasing trend of 0.022 °C/a. Compared with the 1980s, the annual average temperature in the 2000s increased by 0.67 °C. The mean precipitation showed a slight but insignificant increasing trend of 2.779 mm/a (*p* > 0.05). This indicates that the climate was warmer and wetter in the SRB.

**Table 1:** Trend analysis of annual precipitation and temperature using the M–K test in the SRB.

Index	Trend test		Significance
	Trend rate	*Z* values	
Temperature	0.022 °C/a	2.75	0.01
Precipitation	2.779 mm/a	0.37	–

### Temporal and spatial distribution of BW and GW

#### Temporal variation of BW and GW

**Figure 3:**
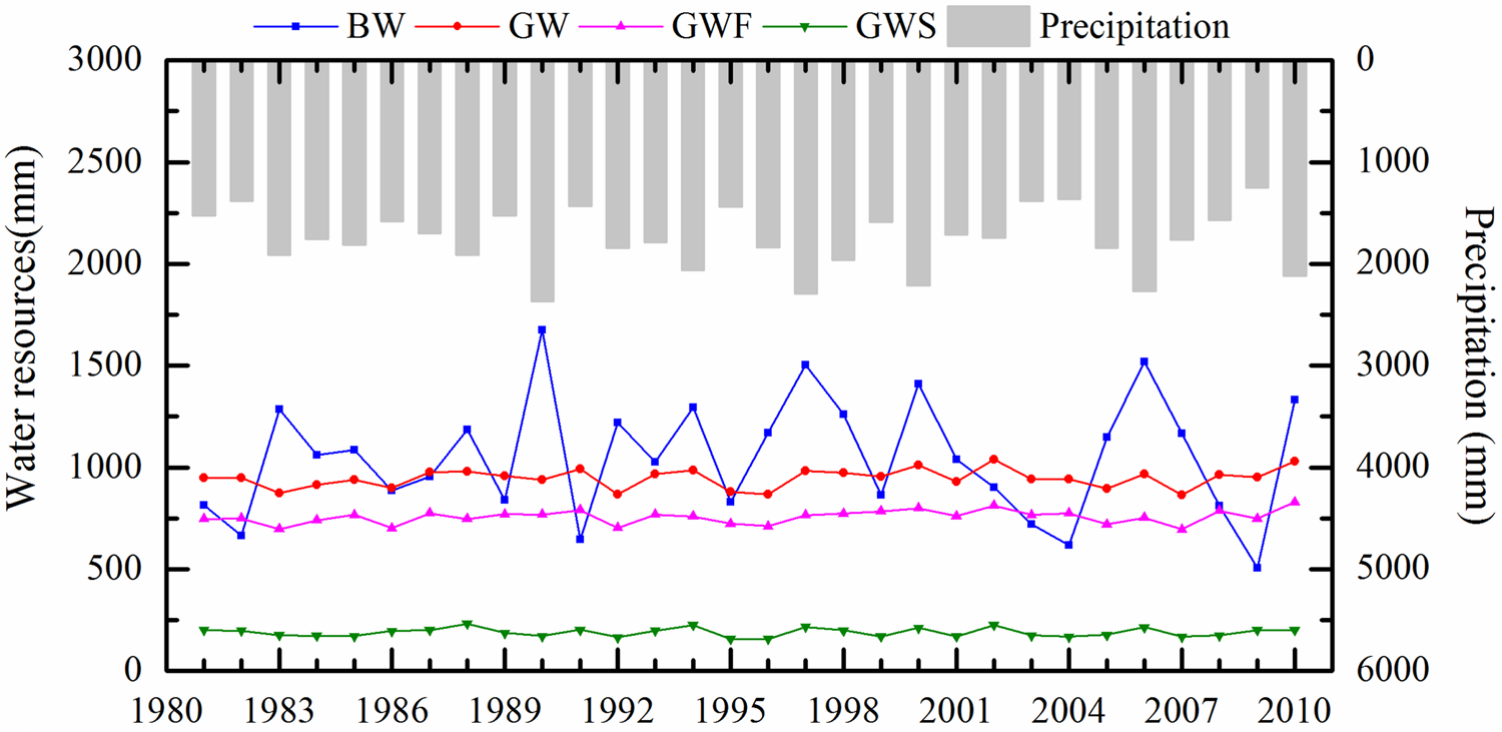
Annual variation of BW and GW resources in the SRB for 1981–2010.

The BW and GW resources in the SRB were calculated based on the calibrated SWAT model, as shown in Fig. 3. From 1981 to 2010, the annual average BW content was 1049 mm, accounting for 52.6% of the total water resources. The interannual variation in BW resources fluctuated dramatically, ranging from 506 to 1674 mm. The annual average GW content was 946 mm, accounting for 47.4% of the total water resources. The interannual difference in GW was relatively small, varying from 864 to 1039 mm. BW, GWF, and GWS accounted for 34.76–64.06%, 29.38–51.40%, and 6.56–13.84% of the total water resources, respectively. The temporal variation in BW was nearly consistent with the precipitation trend. The maximum precipitation occurred in 1990, and the maximum BW appeared simultaneously. Moreover, the precipitation in the dry year of 2009 was the minimum along with the BW resource.

As shown in Table 2, there was a slight upward trend in BW from 1980 to 2010, with a rate of change of 1.569 mm/a. However, the increasing trend was insignificant, and the Z value was 0.11. The GWF decreased in the 1980s and increased in the 1990s. The change tendency rate reached 1.125 mm/a with a Z value of 1.68 (*p* < 0.1). The GWS was relatively stable, with a slight but insignificant decreasing tendency rate of − 0.039 mm/a. In summary, GW resources increased slightly, with a tendency rate of 1.051 mm/a.

**Table 2:** Trend analysis of annual BW and GW resources using the M–K test in the SRB.

Index	Trend test		Significance
	Trend rate (mm/a)	*Z* values	
BW	1.569	0.11	–
GW	1.051	0.89	–
GWF	1.125	1.68	0.1
GWS	− 0.039	− 0.14	–

**Figure 4:**
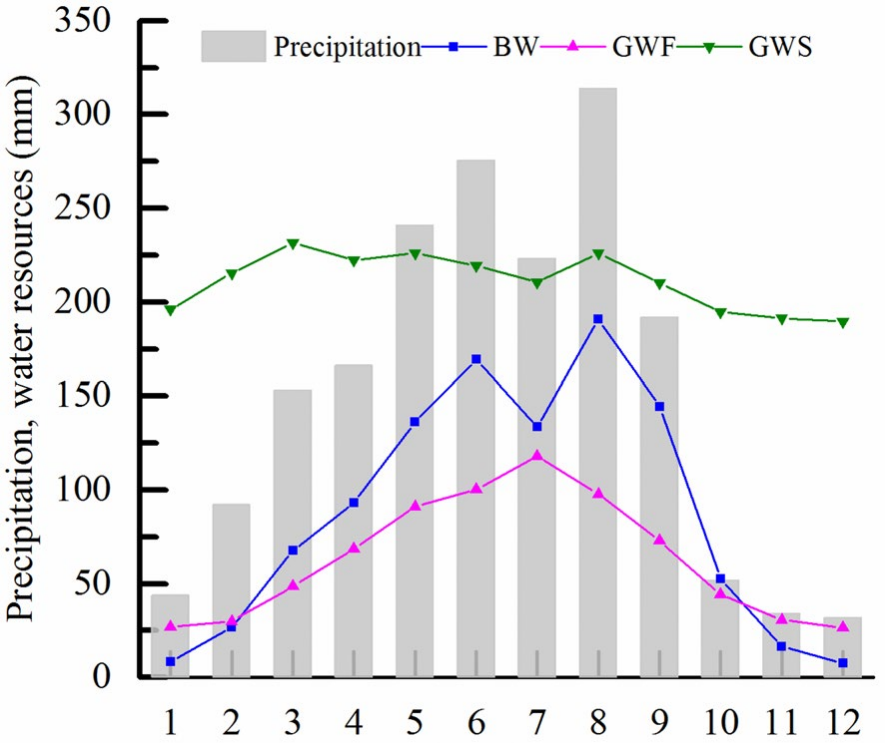
Average monthly variation of BW and GW resources in the SRB.

On a monthly scale, the distribution of BW is very uneven, presenting two peaks in June and August, which is coincident with the precipitation data (Fig. 4). Influenced by temperature and evapotranspiration, the maximum value of GWF occurred in July and summer. The distribution of GWS was relatively even within the year, with the values in spring and summer being slightly more significant than those in autumn and winter. This is related to the abundant precipitation and sufficient soil water supply in spring and summer. During the wet season (April to October), the total amount of BW resources was larger than that of GWF due to higher precipitation. However, during the dry season (November to February), the GWF was greater than the BW. Because of the soil water moisture in the early stage, the GWS is generally greater than the BW and GWF, especially in spring, autumn, and winter.

#### Spatial distribution of BW and GW

**Figure 5:**
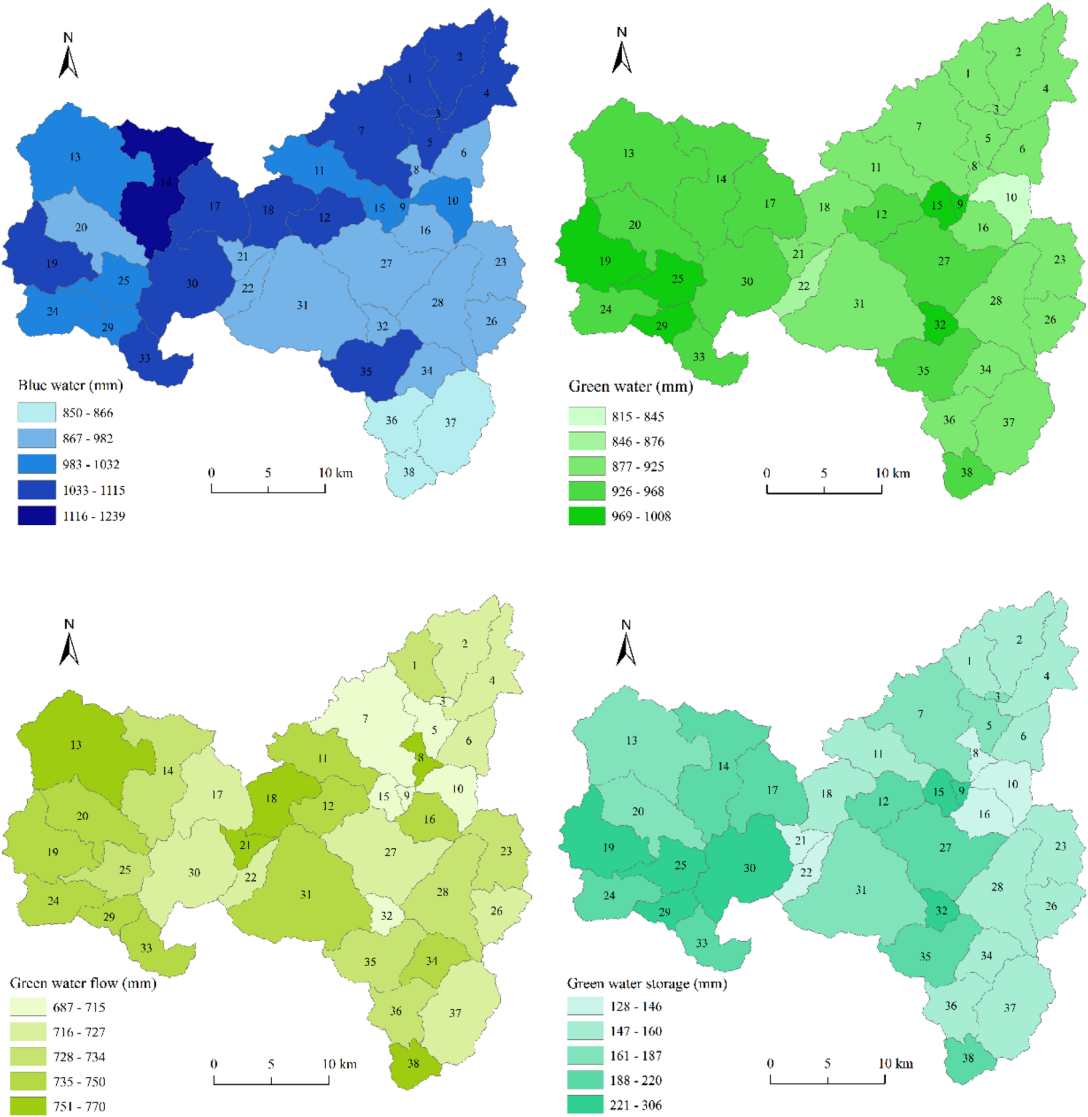
Spatial distribution for BW, GW, GWF, and GWS in the SRB.

There was distinct spatial heterogeneity in the BW and GW resources in the basin (Fig. 5). The average annual BW content ranged from 850 to 1239 mm, with a coefficient of variation (CV) of only 0.080. BW resources were high, with an average value of 1103 mm in subbasins 14, 19, 30, and 33 (located in the upper and middle reaches of the Taoxi River) and subbasins 1, 2, 3, 4, 5, and 7 (located in the upper reaches of the Huyangxi River). In contrast, the BW resources in the lower reaches of the Taoxi and Huyangxi Rivers were relatively low, especially in the subbasins 36, 37, and 38 around the Shanmei Reservoir (with an average value of 858 mm). The BW resources showed a decreasing trend from the upper to the lower reaches.

Compared to the average annual BW content, the GW content ranged from 815 to 1008 mm, with a CV value of 0.048. GW resources were high, with an average value of 980 mm in subbasins 19, 25, and 29 (located in the upper and middle reaches of the Taoxi River) and subbasins 9, 15, 27, and 32 (located in the middle and lower reaches of Huyangxi River). A lower amount of GW resources occurred in subbasins 10 and 22 (837 mm). The spatial difference in the GWF was very small, with a CV value of 0.023. In comparison, the spatial differentiation of the GWS was more significant, with a CV value of 0.232.

### BW and GW output with different land cover

**Figure 6:**
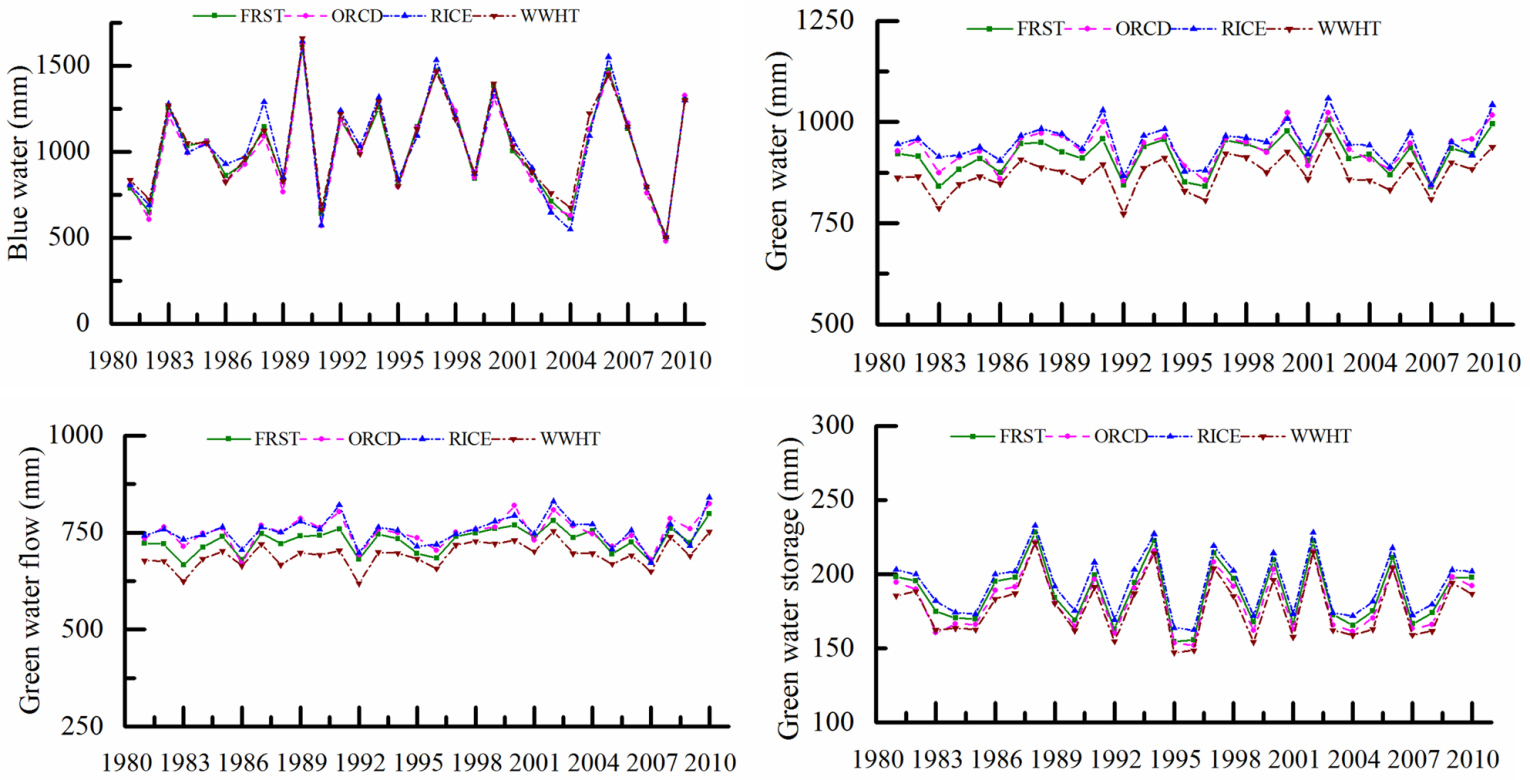
BW and GW resources for different land use classes.

The difference in BW with different land-use covers was rather small during 1981–2010 (Fig. 6). The annual average BW content of the forest land was 1022 mm, whereas that of paddy fields and rainfed croplands reached 1037 mm. The BW resource in the forest area was slightly less due to the interception effect of forest on rainfall. The GWF of the paddy field was also the largest (754 mm), which was followed by that of orchards (751 mm); however, the forest and rainfed croplands exhibited values of 730 mm and 693 mm, respectively. The characteristics of GWS per unit area are similar to those of GWF, with the largest value being observed in paddy fields (192 mm). The GWS of forests (187 mm) is greater than that of orchards (181 mm) and rainfed croplands (178 mm), reflecting the forest’s capacity for soil water conservation.

## Discussion

### Composition and characteristics of BW and GW

Overall, BW resources in the SRB were more abundant, and the annual average BW content accounted for 52.6% of the total water resources. In addition, the number of GW resources was slightly lower, accounting for 47.4% of the water resources. On a global scale, BW accounts for one-third of water resources. Meanwhile, the remaining two-thirds are mainly GW resources, including soil water stored in the unsaturated layer and GWF exhibiting evapotranspiration. However, most studies have shown that the distribution proportions of BW and GW in different climate regions around the world are quite different, indicating regional characteristics^1,16,20,21,33,34^ (Table 3).

**Table 3:** Comparisons of annual average values or ranges of BW (mm) and GW (mm) at different climate basins around the world.

Basin	Country	Climate	BW	GW
Shanmei reservoir basin	China	Subtropical monsoon climate	1049	946
The upper Narmada basin^21^	India	Tropical monsoon climate	1022	731
Bazoft watershed^20^	Iran	Subtropical mediterranean climate	400–1300	79–278
Ohi river basin^1^	United States	Temperate continental climate	250–720	760–960
Guder river basin^33^	Ethiopia	Tropical savanna climate	529–564	878–898
Kashafrood basin^34^	Iran	Subtropical continental semi-arid climate	37.6	255.6
Yellow river basin^16^	China	temperate continental climate	150	373

Owing to their large aridity index in arid areas, more precipitation is lost due to evapotranspiration, resulting in relatively less surface runoff. GW resources account for a large proportion of the total water resources, which can reach 60–85%^35–38^, thereby becoming the main water resource in arid areas. In comparison, owing to the abundant precipitation and high runoff coefficient in humid areas, the proportion of BW in the total water resources can generally exceed that in arid areas. Chen et al.^36^ studied ten major river basins in China and found that in the Zhejiang Fujian basin, the BW coefficient is generally between 0.5 and 0.55, and the GW coefficient is between 0.45 and 0.5; these results are consistent with the findings of this study. According to Chen et al.^36^, the BW coefficient is generally in the range of 0.5–0.55, and the GW coefficient is in the range of 0.45–0.5 in the Zhemin river; these results are also consistent with the findings of our study.

Although the SRB is located in a humid region and BW occupies a more significant proportion of the water resources, temperature has shown a significant upward trend since 1981. Therefore, the evapotranspiration rate further increased, and the proportion of GWF in the total water resources increased from 37.64% in the 1980s to 39.64% in the 2000 s. In the future, from 2071 to 2100, GWF in humid regions may increase significantly. Compared with the period from 1971 to 2000, the GW resources in the Zhemin River may increase by 22–27%^36^, indicating that GW resources will play a prominent role in future ecosystems. GW and BW resources are interlinked, and changes in GW also drive changes in BW ^9^. Most studies have shown that global temperatures will exhibit a continuous upward trend in the future^39,40^. With global warming, evapotranspiration changes related to GW may affect runoff generation, BW consumption^41,42^, and availability of water resources in downstream areas. Thus, it is necessary to carry out reasonable allocation of BW and GW resources, while meeting the agricultural and domestic water demands for the long term.

### Effect of climate variability on BW and GW

Previous studies have shown that precipitation and temperature are two important environmental factors that affect BW and GW resources^43,44^. Zhao et al.^24^ found that in recent decades, BW and GW resources in the Weihe River basin have decreased mainly because of the reduction in precipitation. Since 1980, precipitation in the SRB has shown a weak and insignificant uptrend. With increased precipitation, a large proportion of rainwater contributes to surface runoff and lateral flow, which eventually become BW resources. Furthermore, a small part of rainwater infiltrates the soil to form GWS. Nevertheless, soil water mainly depends on antecedent soil moisture, which is less affected by precipitation and fluctuates more gently. The temporal variation of BW is coincident with precipitation, and their correlation coefficients at annual and monthly scales can reach 0.98 and 0.968, respectively (*p* < 0.01) (Table 4).

**Table 4:** Correlations between climate conditions and blue and GW resources on annual and monthly scales.

Index	Annual		Monthly	
	Precipitation	Temperature	Precipitation	Temperature
BW	0.980**	0.12	0.968**	0.895**
GW	0.205	0.408*	0.966**	0.932**
GWF	0.096	0.398*	0.915**	0.906**
GWS	0.295	0.158	0.783**	0.351

Therefore, owing to the significant effect of precipitation, BW resources have also shown a weak uptrend since 1981. Due to the synchronization of rain and heat in the SRB, the temperature was significantly related to the BW on a monthly scale, with an *r* value of 0.895 (*p* < 0.01). However, there was no significant correlation between the temperature and BW on an annual scale.

The GWF is very sensitive to temperature, exhibiting significant correlation coefficients of 0.398 (*p* < 0.05) and 0.906 (*p* < 0.01) on annual and monthly scales, respectively. With global warming, the temperature in the SRB also showed an upward trend, which strengthened the evapotranspiration intensity of surface water, soil, and vegetation^45^, resulting in a significant upward trend in GWF (*p* < 0.1). It has also been reported that the GWF will continue to increase with further warming in the future^20,21^. However, precipitation and temperature did not affect GWS on an annual scale in the SRB. There was no significant correlation between GWS and monthly temperature, while significant relationships were observed between monthly precipitation and GWS (*r* = 0.783; *p* < 0.01). GWS refers to water stored in the unsaturated soil layer. Therefore, sufficient infiltration during the flood season also supplements the soil water moisture^16^.

Several studies have also concluded that although climate variability affects both BW and GW, precipitation has a greater impact on BW resources on both annual and monthly scales^46,47^; this finding is consistent with the conclusions of our study. GW resources are less susceptible to variations in annual precipitation, whereas they are more closely related to temperature^1,21^. However, these studies have mainly focused on the relationship of climate variability with BW and GW resources on an annual scale. Our study found that GWS was also significantly related to precipitation on a monthly scale but not to temperature, which is different from the characteristics of GWF. With the comprehensive effects of GWF and GWS, GW resources also showed significant correlations with monthly precipitation and temperature (*p* < 0.01). This indicates that the relationships between climate conditions and different components of GW resources may vary at different time scales.

**Figure 7:**
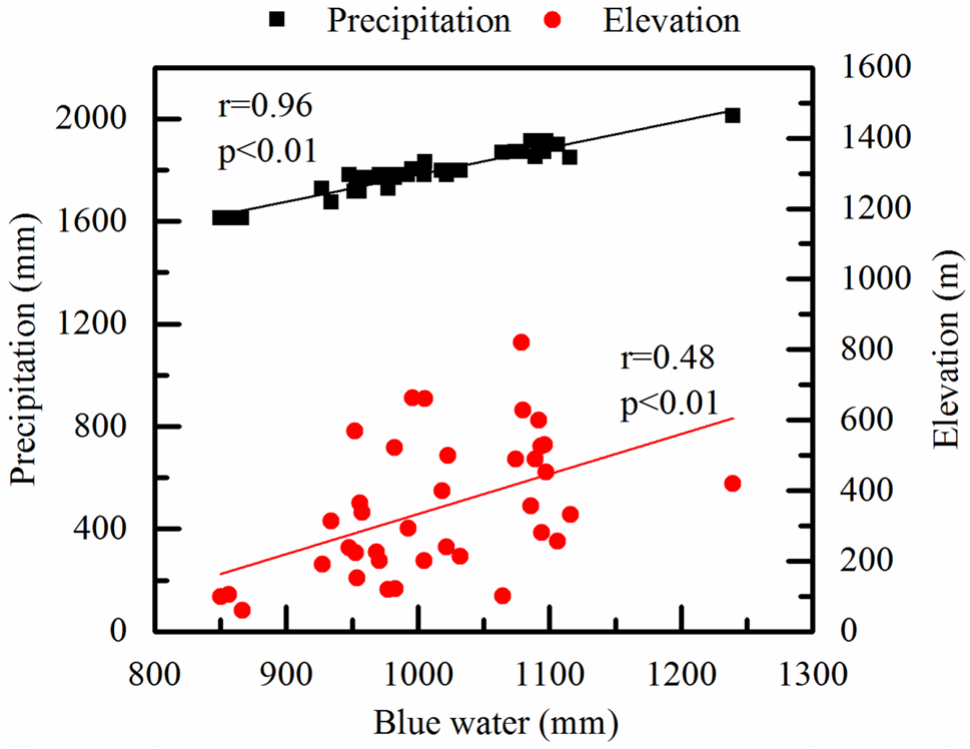
Correlations between BW and precipitation, elevation in the SRB.

The spatial distribution of BW resources in the SRB was significantly affected by precipitation patterns, with a correlation coefficient of 0.961 (*p* < 0.01) (Fig. 7). However, there was no significant correlation between the spatial distribution of precipitation and the GW resources. Furthermore, the distribution of precipitation also depends on elevation. The altitudes of the upper reaches of the Tao and Huyang Rivers were more than 600 m. In contrast, the altitudes of the subbasin 36, 37 and 38 where Shanmei Reservoir is located were less than 300 m. Therefore, the spatial distribution of BW showed a decreasing pattern from the upper reaches to the downstream reaches, which was closely related to the terrain altitude distribution (*r* = 0.48, *p* < 0.01) (Fig. 7).

### Effect of land use types on BW and GW

**Figure 8:**
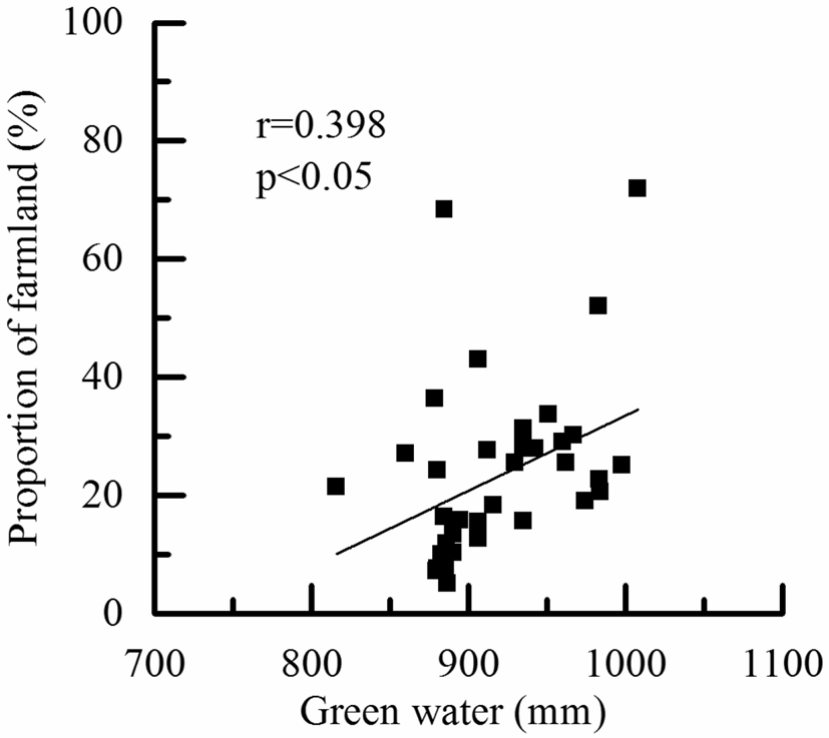
Correlations between GW and proportion of farmland in the SRB.

Compared with BW, the spatial distribution of GW resources is more affected by land use than precipitation^1^. The proportion of farmland in the subbasin affected GW resources and had a correlation coefficient of 0.398 (*p* < 0.05) (Fig. 8). Paddy fields, rainfed croplands, and orchards were the main farmland types in the SRB. Affected by crop growth and irrigation, crop evapotranspiration and soil water storage are greater in sub-basins dominated by farmland, resulting in excellent GW resources. For example, the proportion of farmland in sub-basins 9 and 15 reaches 71.85% and 52.08%, respectively, and their annual average GW content can reach 1008 mm and 983 mm, respectively. Overall, the spatial distribution of BW resources is related to climate variability, and land-use types lead to the spatial differentiation of GW resources^35^. Compared with precipitation, the influence of land cover on hydrological processes is more complicated. The distribution characteristics of land cover determine the distribution patterns of evapotranspiration, thus affecting the spatial differentiation of green and BW resources^48,49^.

The GWF of paddy fields is larger than that of forests in the SRB, mainly because different land covers have varying effects on evapotranspiration. The paddy field has a large water area and sufficient water supply, and the corresponding evapotranspiration is also greater than that of other land cover types. As the main component of GW resources, evapotranspiration determines the quantity of GWF. It has been reported that an increase in farmland irrigation area can reduce the amount of BW and increase GWF^12,26^. Mao et al.^35^ found that the GW coefficient of farmland was greater than that of forest and grassland, and the evapotranspiration of farmland was also higher than that of forest and grassland. When crops are in the growing season, with high temperature and sufficient irrigation water, the double effect of plant transpiration and evaporation of soil and water increases evapotranspiration. Liang et al.^15^ found that most crops are shallow-rooted plants and are sensitive to high evapotranspiration environments after farmland growth, which leads to an increase in soil water retention capacity and soil evaporation.

The GWS in paddy rice is also larger than that in other land use types in the SRB, mainly because of the high soil water content. Forest land also has a larger amount of GWS than that of orchards and rainfed cropland, which may be related to the forest’s ability to conserve soil water. Qiu et al.^50^ and Zhao et al.^24^ showed that after the implementation of returning farmland to forest and grassland, the BW and GWF in the basin decreased, while the soil water content (GWS) increased. A large amount of forest can intercept more precipitation and increase soil infiltration^51^, thus increasing GWS and reducing BW in the flood season, which is very important for vegetation restoration.

Land use types are commonly responsible for the spatial distribution of evapotranspiration and root soil water (GW), and climatic variability has a greater impact on water yield and deep aquifer recharge (BW). Similar results showing the relationship of land use with GW and that of climate with BW were obtained by Zhao et al.^24^. However, other studies have shown that the impact of land use change on annual total evaporation is not significant. The variation in BW is greater than that in GW due to land use change^25,26^. Therefore, there is insufficient evidence to show universal causality between climate variability, land use change, and transformation of BW to GW.

## Conclusions

The temporal and spatial distributions of BW and GW in the SRB located in southeast China were simulated using the SWAT model, and the influences of climate variability and land use on BW and GW were analyzed. The results indicated that the annual average BW content was 1049 mm, accounting for majority of the total water resources in the SRB (52.57%); meanwhile, annual average GW content accounted for 47.43% of the water resources, with an average value of 946 mm. From 1980 to 2010, the annual average temperature in SRB showed an obvious increasing trend of 0.22 °C/10a (*p* < 0.01), while the precipitation increased insignificantly. BW also showed a slight upward trend, which was consistent with precipitation. The GWS was relatively stable, whereas the GWF showed an increasing trend, with a tendency rate of 1.125 mm/a (*p* < 0.1). The distribution of BW decreased from upstream to downstream, and the spatial variability of GWS was greater than that of BW and GWF. The BW and GWF of paddy fields was greater than that of forest land, while the GWS of forest was greater than that of orchard and rainfed cropland; these results indicated that forests can hold more soil water and reduce the BW content in the flood season. Precipitation is a key factor affecting the temporal variation in BW, with significant correlations at annual and monthly scales. GWF is more sensitive to temperature at annual and monthly scales. GWS is only correlated with precipitation on a monthly scale; however, it is not related to precipitation or temperature on an annual scale. The spatial distribution of GW is significantly associated with the proportion of farmland within the subbasins. The variation in land use types leads to spatial differentiation of GW resources.

In general, this study provides significant insights into the fresh water availability in terms of BW and GW. The information of relative impacts of climate and land use on the dynamics of BW and GW, as quantified in this study, is very helpful for decision making on water resources management, protection, and the national policy makers considering the global warming and regional human activities.

## Data Availability

The data that support the findings of this study are available from the China Meteorological Administration and Administration Office of Shanmei Reservoir but restrictions apply to the availability of these data, which were used under license for the current study, and so are not publicly available. Data are however available from the authors upon reasonable request.
